# Relationship between hyponatremia at hospital admission and cardiopulmonary profile at follow-up in patients with SARS-CoV-2 (COVID-19) infection

**DOI:** 10.1007/s40618-022-01938-9

**Published:** 2022-10-25

**Authors:** D. Malandrino, A. Berni, B. Fibbi, B. Borellini, D. Cozzi, D. Norello, F. Fattirolli, F. Lavorini, I. Olivotto, C. Fumagalli, C. Zocchi, L. Tassetti, L. Gozzi, N. Marchionni, M. Maggi, A. Peri, the AOU Careggi COVID-19 Follow-up Study Group, Michele Spinicci, Lorenzo Zammarchi, Leonardo Gori, Guja Bernacchi, Lorenzo Ciambellotti, Iacopo Vellere, Matteo Vannini, Sara Montali, Francesco Tonarelli, Viola Camartini, Giada Turrin, Giuseppe Dario Testa, Simona Virciglio, Enrico Gardellini, Carolina Corsi, Sofia Espinoza Tofalos, Rossella Marcucci, Laura Rasero, Lorenzo Giovannoni, Luca Livi, Maria Vittoria Silverii

**Affiliations:** 1 Department of Experimental and Clinical Medicine, University of Florence, Florence, Italy; 2 Internal Medicine Unit 3, Careggi University Hospital, Florence, Italy; 3 Endocrinology Unit, Careggi University Hospital, Florence, Italy; 4 Pituitary Diseases and Sodium Alterations Unit, Careggi University Hospital, Florence, Italy; 5 Department of Experimental and Clinical Biomedical Sciences “Mario Serio”, University of Florence, Viale Pieraccini, 6, 50139 Florence, Italy; 6 Radiology Emergency Department, Careggi University Hospital, Florence, Italy; 7 Cardiac Rehabilitation Unit, Azienda Ospedaliero-Universitaria Careggi, Florence, Italy; 8 Cardiomyopathy Unit, Careggi University Hospital, Florence, Italy

**Keywords:** SARS-CoV-2 (COVID-19) infection, Hyponatremia, SIAD, Pneumonia, Outcome, Follow-up

## Abstract

**Purpose:**

Hyponatremia occurs in about 30% of patients with pneumonia, including those with SARS-CoV-2 (COVID-19) infection. Hyponatremia predicts a worse outcome in several pathologic conditions and in COVID-19 has been associated with a higher risk of non-invasive ventilation, ICU transfer and death. The main objective of this study was to determine whether early hyponatremia is also a predictor of long-term sequelae at follow-up.

**Methods:**

In this observational study, we collected 6-month follow-up data from 189 laboratory-confirmed COVID-19 patients previously admitted to a University Hospital. About 25% of the patients (*n* = 47) had hyponatremia at the time of hospital admission.

**Results:**

Serum [Na^+^] was significantly increased in the whole group of 189 patients at 6 months, compared to the value at hospital admission (141.4 ± 2.2 vs 137 ± 3.5 mEq/L, *p* < 0.001). In addition, IL-6 levels decreased and the PaO_2_/FiO_2_ increased. Accordingly, pulmonary involvement, evaluated at the chest X-ray by the RALE score, decreased. However, in patients with hyponatremia at hospital admission, higher levels of LDH, fibrinogen, troponin T and NT-ProBNP were detected at follow-up, compared to patients with normonatremia at admission. In addition, hyponatremia at admission was associated with worse echocardiography parameters related to right ventricular function, together with a higher RALE score.

**Conclusion:**

These results suggest that early hyponatremia in COVID-19 patients is associated with the presence of laboratory and imaging parameters indicating a greater pulmonary and right-sided heart involvement at follow-up.

**Supplementary Information:**

The online version contains supplementary material available at 10.1007/s40618-022-01938-9.

## Introduction

Hyponatremia, defined as a serum sodium concentration ([Na^+^]) < 135 mEq/L, is the most common electrolyte disorder detected in hospitalized patients [1]. It occurs in inpatients cohorts with a prevalence of about 30%, which rises up to 42% in intensive care settings [2, 3]. In hospital settings, hyponatremia may occur due to several diseases. The syndrome of inappropriate antidiuresis (SIAD) accounts for 40–50% of cases, with even higher prevalence in pneumonia, subarachnoid hemorrhage and traumatic brain injury [4]. In the remaining cases of hyponatremia in hospitalized patients, hypovolemic (e.g., vomiting, diarrhea, sodium-losing nephropathies) or hypervolemic (e.g., liver, heart or kidney failure) etiologies may be present.

If prolonged over time, the perturbation of internal homeostasis due to even mildly reduced serum [Na^+^] can lead to a permanent damage and a clear association between hyponatremia and increased morbidity and mortality have been demonstrated [5–8]. In this view, a prompt and appropriate correction of this electrolyte imbalance is critical to prevent short- and long-term complications, as suggested by the reversibility of clinical abnormalities secondary to mild or moderate chronic hyponatremia and the reduction of mortality after low serum [Na^+^] reversal [9–11].

Not surprisingly, hyponatremia is detected in about 20–30% of patients hospitalized for SARS-CoV-2 (COVID-19) infection and is mostly due to SIAD or hypovolemia [12]. A close association between hyponatremia, COVID-19 and a more frequent unfavorable outcome has been described [13–16]. By a retrospective analysis of COVID-19 patients during the first pandemic period, we previously demonstrated a prevalence of 22.9% of patients with hyponatremia at hospital admission. These patients had a worse respiratory performance and higher interleukin 6 (IL-6) levels compared to normonatremic ones. Moreover, we identified hyponatremia as an independent predictor of in-hospital mortality (2.7-fold increase vs normonatremia) in COVID-19 patients, with a 14.4% increased risk of death for each mEq/L of serum [Na^+^] reduction [16]*.* These findings were also supported by two systematic reviews and a meta-analysis of 8 studies and 11,493 patients, which showed that the presence of hyponatremia increased the probability of a poor outcome in COVID-19 patients [17, 18].

It has been shown that IL-6, one of the most important cytokines involved in hyperinflammation syndrome and COVID-19-induced pathology [19], induced vasopressin secretion both by a direct hypothalamic stimulation and by inducing alveolar basement membrane injury and pulmonary hypoxia and vasoconstriction [20–22]. Thus, we hypothesized that IL-6 might represent the common denominator of both acute respiratory failure and SIAD-related hyponatremia in COVID-19 patients [16].

Although most of COVID-19 patients completely recover within 4 weeks after the onset of acute infection, several reports described a multitude of prolonged or recurrent symptoms after negative nasopharyngeal swab test with a prevalence ranging from 35 to 87% in different cohorts [23–26]. This condition, characterized by the presence of different physical and/or neuropsychiatric symptoms for at least 12 weeks without an alternative explanation, is defined as post-COVID or long-COVID syndrome.

We questioned whether hyponatremia observed in patients hospitalized for COVID-19 at admission might be associated not only with a short term more severe outcome [16], but also with a different clinical profile at follow-up. Therefore, we examined the clinical charts of hyponatremic and normonatremic patients included in our previous study, who were systematically followed up after COVID-19 at the Careggi University Hospital in Florence, Italy.

## Materials and methods

### Follow-up design

**Fig. 1:**
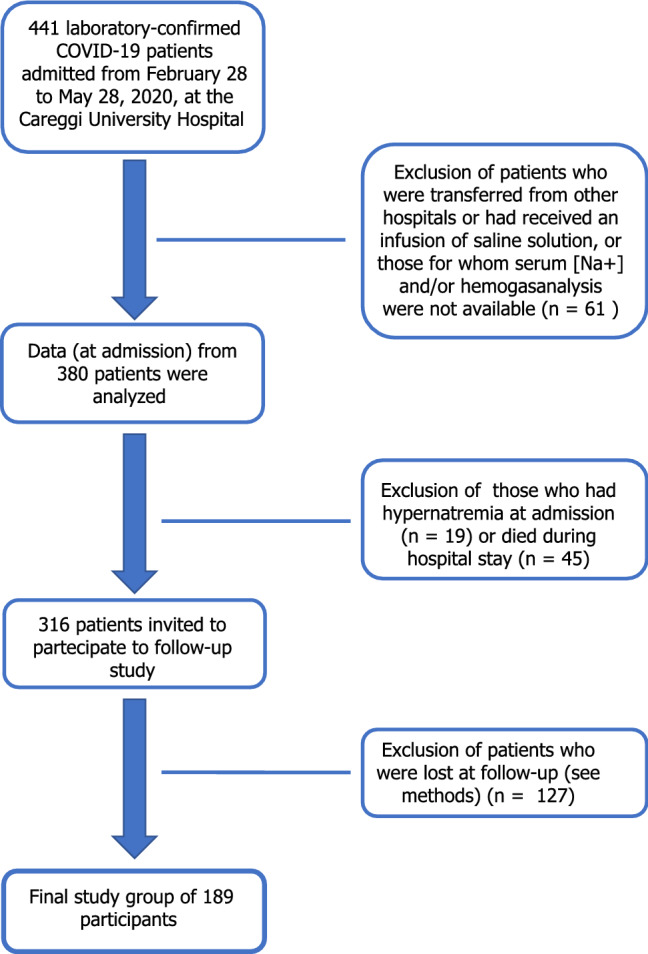
Flowchart of the study design

The AOU Careggi COVID-19 Follow-up study Group is a longitudinal, prospective, single-center, cohort follow-up project. All consecutive patients admitted to the Careggi University Hospital in Florence (Italy) for COVID-19 were prospectively enrolled in a clinical and instrumental follow-up program aimed at monitoring symptoms at 6, 9 and 12 months and at evaluating cardiovascular, respiratory, infectious, and functional status at 6 months [26, 27]. The study was conducted in accordance with the guidelines of the Declaration of Helsinki and its protocol approved by the local Ethical Review Board (Comitato Area Vasta Centro, CARE-COVID19 AOU Careggi Protocol 00/08761 April 2020). Of the 380 patients, admitted to the hospital from February 28 to May 28 2020 and included in our previous observational study [16], those who had hypernatremia at admission (*n* = 19), died during hospital stay (*n* = 45) or were lost during follow-up (i.e., patients with motor disability, cognitive impairment, or admission to long-term care facilities; *n* = 127), were excluded from the present study (Fig. 1).

Briefly, all patients discharged from a COVID-19 Unit received a phone call after 2 weeks and were invited to participate in the follow-up program. Each patient provided an informed consent. Each patient was screened for symptoms and was re-directed to multidisciplinary evaluation, which included a routine blood laboratory assessment (a complete list of exams is presented in Supplementary Table 1), arterial blood gas analysis, chest X-ray, cardiovascular (EKG, transthoracic echocardiography) and respiratory evaluation (pulmonary function tests, PFTs; 6 min walking test).

### RALE score evaluation

The radiographic assessment of lung edema (RALE) score is a widely accepted and highly reproducible numeric scoring system recently developed to assess the degree of lung edema in acute respiratory distress syndrome (ARDS) [28]. Likewise, during the COVID19 pandemic, this radiographic assessment was also found to be a useful and reliable tool in predicting the severity of lung involvement and adverse outcomes in patients with SARS-CoV-2 pneumonia [29, 30].

Briefly, to determine the RALE score, the chest at chest-X-ray is divided into four quadrants to quantify the extent of alveolar opacities and consolidation. To calculate the final RALE score (ranging from 0 to 48, which expresses dense consolidation in > 75% of each quadrant), two experienced radiologists, both blinded to patients’ basal serum [Na^+^], independently evaluated all chest X-ray examinations (performed at admission and at 6-month visit).

Data were stored on a dedicated online password-protected platform, which was only accessible to the healthcare staff involved in the follow-up program.

### Statistical analysis

Statistical analysis was performed with the IBM SPSS 28 system (SPSS Inc., Chicago, IL, USA). Continuous data were expressed as mean (± SD) and as median (with interquartile ranges) where appropriate, while categorical variables are described as the number (percentage). The *t* test or Mann–Whitney *U* test (in case of values with a skewed distribution) were used to compare continuous variables. Chi-squared and Fisher’s exact tests were used to compare proportions for categorical variable. Two-tailed comparisons with a *p* value < 0.05 were considered statistically significant. Serum [Na^+^] was adjusted for serum glucose concentration as previously described [16]. Univariate linear regression analysis (Pearson correlation coefficient) was used to test the relation between P/F and RALE score at admission and the correlation coefficient *r*. Logistic regression (with Odds Ratio, OR, and 95% confidence intervals, 95% CI) was used to identify the possible relationship between RALE scores (at admission and at follow-up chest X-ray) and the diagnosis of hyponatremia at admission. Adjusted models included age, follow-up time (months) and serum IL-6 levels, as specified.

## Results

### Evaluation at hospital admission

The final study group consisted of 189 participants (61.4% men, mean age 62.5 ± 13.9 years), who completed the 6-month follow-up examinations. Forty-seven patients (24.9%) showed hyponatremia and 142 (75.1%) showed normal serum [Na^+^] at admission (hereafter indicated as basal hypoNa and basal normoNa, respectively). The baseline characteristics and clinical outcomes of the participants are reported in Table 1.

**Table 1:** Demographic and clinical characteristics of the patients

Variables	All (*n* = 189)	NormoNa (*n* = 142)	HypoNa (*n* = 47)	*p*
Age (years)	62.5 ± 13.9	61.8 ± 13.9	64.6 ± 13.7	0.226
Male, *n* (%)	116 [61.4%]	87 [61.3]	29 [61.7]	0.958
Hospitalization (days)	16.4 ± 14.8	14.9 ± 13.1	20.9 ± 8.5	0.015
Age (years)	62.5 ± 13.9	61.8 ± 13.9	64.6 ± 13.7	0.226
Serum [Na^+^] (mEq/L)	137.0 ± 3.5	138.5 ± 2.3	132.3 ± 2.3	< 0.001
Serum glucose (mg/dL)	124.7 ± 46.3	123.8 ± 45.0	127.3 ± 50.4	0.679
Serum creatinine (mg/dL)	0.89 [0.72–1.17]	0.89 [0.69–1.18]	0.92 [0.73–1.20]	0.875
BUN (mg/dL)	14.9 [14.0–23.4]	17.3 [14.0–18.7]	14.0 [14.0–18.7]	0.969
P/F (mmHg)	317.3 ± 81.2	328.5 ± 79.9	283.5 ± 76.5	< 0.001
IL-6 (pg/mL)	12.3 [5.1–26.6]	9.3 [5.5–21.0]	17.3 [13.8–34.6]	< 0.001
RALE score	4.0 [2.0–12.0]	4.0 [2.0–12.0]	7.0 [4.0- 20.0]	0.008
NIV, *n* (%)	51 (27.0)	32 (22.5)	19 (40.4)	0.017
ICU transfer, *n* (%)	48 (25.4)	31 (21.8)	17 (36.2)	0.048

Data are expressed as mean (± SD) or median (interquartile range)

*BUN* blood urea nitrogen, *P/F* PaO2/FiO2 ratio, *NIV* non-invasive ventilation

During hospital stay, significant differences were observed between the two groups with a significant higher use of non-invasive ventilation (NIV) (40.4% vs 22.5% patients, *p* = 0.017), length of stay in the hospital (20.9 ± 8.5 vs 14.9 ± 13.1 days, *p* = 0.015) and transfer into ICU (36.2% vs 21.8% patients, *p* = 0.048) in the hyponatremic group. Compared to normonatremic patients, hyponatremic patients had higher IL-6 levels and lower PaO_2_/FiO_2_ (P/F) ratio at admission, whereas no differences in serum creatinine and blood urea nitrogen (BUN) levels were observed.

### Evaluation at follow-up

At 6-month follow-up visit of the whole population of 189 patients displayed higher median values of serum [Na^+^] and P/F ratio, and lower serum IL-6 levels were detected (Table 2) when compared to the corresponding baseline (i.e., at hospital admission) values, together with a significantly lower median RALE score.

**Table 2:** Comparative assessment of laboratory, hemogasanalysis, and chest X-ray findings in study subjects between admission and follow-up visit

Variables	At admission (*n* = 189)	Follow-up (*n* = 189)	*p*
Serum [Na^+^] (mEq/L)	137.0 ± 3.5	141.4 ± 2.2	< 0.001
Serum creatinine (mg/dL)	0.89 [0.72–1.17]	0.89 [0.74–1.11]	NS
IL-6 (pg/mL)	12–3 [5.1–26.6]	2.0 [0.6–6.2]	< 0.001
P/F (mmHg)	317.3 ± 81.2	328.5 ± 79.9	< 0.001
RALE score	4.0 [2.0–12.0]	0.0 [0.0–2.0]	< 0.001

Data are expressed as mean (± SD) or median (interquartile range)

*P/F* PaO2/FiO2 ratio

### Basal hypoNa vs basal normoNa: laboratory findings at follow-up

Laboratory measurements are summarized in Table 3. The comparison between the two study subgroups (basal hypoNa vs basal normoNa), revealed no differences for the majority of lab parameters, including IL-6 levels. Interestingly, in hypoNa patients, significantly higher values of markers of myocardial injury, such as troponin T and NT-proBNP were observed. Moreover, at the time of follow-up visit, higher LDH and fibrinogen levels were found in patients with basal hypoNa than in those with normoNa.

**Table 3:** Laboratory findings (blood parameters) at 6-month follow-up

Variables	All (*n* = 189)	NormoNa (*n* = 142)	HypoNa (*n* = 47)	*p*
White blood cell (× 10^9^/L)	6.3 [5.2–7.0]	6.3 [5.3–7.0]	6.1 [5.2–7.1]	0.809
Hemoglobin (g/dl)	14.4 [13.1–15.4]	14.4 [13.4–15.4]	14.3 [12.8–15.6]	0.561
Platelet count (× 10^9^/L)	229 [193–269]	230 [193–267]	223 [191–272]	0.812
Creatinine (mg/dL)	0.9 [0.8–1.0]	0.9 [0.8–1.0]	0.9 [0.8–1.1]	0.247
Azotemia (mg/dL)	0.5 [0.3–0.6]	0.5 [0.3–0.6]	0.5 [0.3–0.7]	0.653
Sodium (mEq/L)	141.0 [140–143]	141.0 [140–143]	141.0 [140–142]	0.175
Potassium (mEq/L)	4.2 [4.0–4.4]	4.2 [3.9–4.4]	4.3 [4.1–4.4]	0.135
Magnesium (mEq/L)	2.0 [1.9–2.1]	2.0 [1.9–2.1]	2.0 [1.9–2.1]	0.822
Albumine (mg/dl)	45.3 [43.0–46.8]	45.4 [42.8–46.8]	44.9 [43.0–46.7]	0.642
ALT (IU/L)	16.0 [12.0–23.0]	16.0 [12.0–23.0]	17.5 [12.0–22.0]	0.694
Bilirubin (mg/dL)	0.5 [0.4–0.7]	0.5 [0.4–0.7]	0.4 [0.3–0.7]	0.214
aPTT (sec)	29.7 [28.3–31.5]	29.7 [28.3–31.2]	30.5 [28.7–32.6]	0.224
PT (sec)	11.6 [11.2–12.2]	11.6 [11.1–12.1]	11.8 [11.3–12.5]	0.177
Creatine phosphokinase (IU/L)	90.5 [62.0–136.0]	90.0 [59.0–136.0]	95.0 [76.0–138.0]	0.304
LDH (mU/mL)	185.0 [171.0–209.0]	183.0 [169.0–206.0]	195.0 [175.0–215.0]	0.048
Fibrinogen (mg/dL)	314.0 [262.0–363.5]	311.0 [258.0–356.0]	337.5 [290.0–395.0]	0.036
Ferritin (ng/mL)	130.5 [64.5–236.5]	131.0 [61.0–262.0]	130.0 [68.0–217.0]	0.280
C reactive protein (mg/L)	4.0 [4.0–4.0]	4.0 [4.0–4.0]	4.0 [4.0–4.0]	0.369
Troponin T (ng/L)	9.1 [6.0–14.1]	8.3 [5.6–12.5]	11.9 [7.1–20.7]	0.004
NT-Pro-BNP (pg/mL)	75.0 [31.0–178.0]	63.0 [28.5–139]	122.0 [42.0–245.0]	0.022
D-Dimer (ng/mL)	371 [267–591]	371.5 [269–595]	369 [238–553]	0.700
Antithrombin (%)	101 [93–110]	101 [92–110]	102 [93–111]	0.626
Protein C (%)	120.0 [107–137]	121.0 [106–136]	120.0 [108–137]	0.910
IL-6 (pg/mL)	2.0 [0.8–4.8]	1.8 [0.8–3.5]	3.3 [0.8–7.0]	0.082
IL-8 (pg/mL)	8.0 [3.0–34.0]	11.0 [3.5–35.0]	7.5 [2.0–34.0]	0.581
IL-10 (pg/mL)	0.7 [0.1–1.6]	0.7 [0.1–1.6]	0.8 [0.1–1.5]	0.941
Tumor necrosis factor-α (pg/mL)	0.0 [0.0–1.0]	0.0 [0.0–1.2]	0.0 [0.0–1.0]	0.223

Data are expressed as median (interquartile range)

### Basal hypoNa vs basal normoNa: pulmonary and cardiac assessment

At 6-month follow-up, pulmonary function variables and the distance covered during the 6-min walking test were similar in normonatremic and hyponatremic patients. Similarly, arterial blood gas measurements did not significantly differ between the two groups of patients (Table 4).

**Table 4:** Pulmonary function test parameters and blood gas analysis in study subjects (follow-up visit)

Variables	All (*n* = 189)	NormoNa (*n* = 142)	HypoNa (*n* = 47)	*p*
FEV1 (L)	2.8 [2.3, 3.3]	2.8 [2.2–3.3]	3.0 [2.3, 3.5]	0.593
FEV1 (%)	99.0 [86.0–110.0]	99.0 [87.0–110.0]	102.0 [85.0–111.0]	0.916
FVC (L)	3.7 [2.9–4.2]	3.6 [2.9, 4.2]	3.7 [2.9–4.5]	0.613
FVC (%)	95.0 [87.0–108.5]	95.0 [86.0–109.0]	95.0 [91.0–108.0]	0.852
TLC (L)	5.6 [4.9–7.6]	5.6 [4.9–6.5]	6.0 [4.8–6.8]	0.341
TLC (%)	95.5 [87.0–105.0]	94.0 [86.0–105.0]	97.0 [87.0–105.0]	0.892
DLCO (mmol/min/kPa)	7.2 [5.7–10.3]	7.2 [5.7–10.3]	7.2 [5.8–11.0]	0.786
DLCO (%)	80.5 [69.0–91.0]	81.0 [69.0–90.0]	80.0 [66.0–93.0]	0.774
6MWT (meters)	478.0 [420.0–528.0]	462.0 [400.0–528.0]	497.5 [430.0–544.0]	0.174
6MWT (meters, % predict)	92.0 [83.0–100.0]	92.0 [81.0–100.0]	93.0 [86.0–104.0]	0.550
PaO_2_ (mmHg)	85.5 [78.8–92.2]	86.4 [79.1–92.4]	84.8 [76.6–92.0]	0.248
Pa CO_2_ (mmHg)	39.3 [36.8–41.4]	39.4 [36.9–41.3]	39.3 [36.6–41.6]	0.862
SaO_2_ (%)	97.4 [96.4–97.9]	97.4 [96.5–97.8]	97.2 [96.0–98.0]	0.320
P/F (mmHg)	398 [365.0–432.0]	398.5 [371.0–429.0]	396.0 [364.0–436.0]	0.548
Lactate (mg/dl)	0.8 [0.6–1.2]	0.9 [0.6–1.2]	0.7 [0.6–1.2]	0.315

Data are expressed as median (interquartile range)

*FEV1* forced expiratory volume in one second, *FVC* forced vital capacity, *TLC* total lung capacity, *DLCO* diffusion capacity for carbon monoxide, *6MWT* 6 min walking test, *PaO2* partial pressure of arterial oxygen, *PaCO2* partial pressure of arterial carbon dioxide, *SaO2* oxygen saturation in arterial blood, *P/F* PaO2/FiO2 ratio

Transthoracic echocardiography showed similar left ventricular wall thickness and systolic function, including left ventricular ejection fraction (LVEF) between the two groups. However, patients with basal hypoNa showed significantly worse echocardiographic parameters of right ventricular (RV) systolic function compared to patients with normoNa, with an increased RV dimension, higher RV-right atrium (RA) pressure gradient (∆P RV-RA), higher systolic pulmonary artery pressure (sPAP) and lower tricuspid annular plane systolic excursion (TAPSE) values (Table 5).

**Table 5:** Echocardiographic assessment (follow-up visit)

Variables	All (*n* = 189)	NormoNa (*n* = 142)	HypoNa (*n* = 47)	*p*
IVS (mm)	10.0 [9.0–10.0]	10.0 [8.0–10.0]	10.0 [9.0–11.0]	0.175
LVPW (mm)	9.0 [8.0–10.0]	9.0 [8.0–10.0]	9.0 [8.0–10.0]	0.162
LA (mm)	36.0 [33.0–40.0]	35.0 [33.0–39.0]	39.0 [34.0–42.0]	0.104
LVEF (%)	60.0 [56.0–64.0]	60.0 [57.0–64.0]	60.0 [55.0–64.0]	0.420
AA (mm)	33.0 [30.0–36.0]	33.0 [30.0–35.0]	33.0 [31.0–38.0]	0.053
RV (mm)	34.0 [30.1–38.0]	33.0 [31.0–37.0]	37.5 [32.5–40.0]	0.015
TAPSE (mm)	23.0 [20.0–25.0]	23.0 [20.0–25.0]	21.0 [19.0–25.0]	0.031
sPAP (mmHg)	24.0 [19.0–28.0]	23.0 [19.0–28.0]	25.5 [20.0–31.5]	0.049
∆P RV-RA (mmHg)	18.0 [14.0–23.0]	17.5. [13.0–22.0]	20.0 [15.0–27.0]	0.029

Data are expressed as median (interquartile range)

*LV* left ventricle, *IVS* interventricular septum, *LVPW* left ventricular posterior wall, *LVEF* left ventricular ejection fraction, *LA* left atrium, *AA* ascending aorta, *RV* right ventricle, *RA* right atrium, *TAPSE* tricuspid annular plane systolic excursion, *sPAP* systolic pulmonary artery pressure, *∆P RV-RA* pressure gradient between RV and RA

### Chest X-ray imaging and RALE score

At admission, 172 of the 189 subjects had abnormal chest radiograph findings with a median RALE score of 7 in the basal hypoNa group and 4 in the basal normoNa group (*p* < 0.001) (Table 1).

**Fig. 2:**
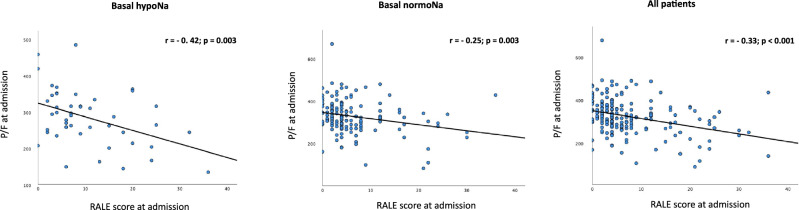
Linear regression for P/F ratio as a function of RALE score at admission in hyponatremic patients, normonatremic patients and in the total group of patients. P/F: PaO2/FiO2 ratio

In the total study group, RALE scores at admission were significantly associated with a worse respiratory gas exchange, as shown in the linear regression model for variation in P/F ratio as a function of RALE scores (Fig. 2). In particular, this association was observed among hyponatremic patients with a tighter inverse correlation between RALE scores and P/F ratio at admission.

After adjustment for age, diagnosis of hyponatremia and serum IL-6 levels was associated with a worse lung involvement at admission (expressed by a RALE score 5) at multivariable logistic regression analysis (*p* = 0.014) (Table 6). Abnormal chest X-ray findings, defined by a RALE score 1, were still present at 6-month evaluation in 49/189 considering the population as a whole; notably, radiographic abnormalities were detected in 18/47 patients of the hypoNa group and in 31/142 patients of the normoNa group (38 vs 21%, respectively; *p* = 0.026).

**Table 6:** Multivariable logistic regression analysis of factors associated with a RALE score at admission ≥ 5

	RALE ≥ 5 OR (95% CI)	*p*	RALE ≥ 5 OR (95% CI)	*p*
Hyponatremia	3.56 (1.72–7.32)	**< 0.001**	2.69 (1.25–1.31)	0.014
IL-6 levels (pg/mL)			1.16 (1.01–1.33)	0.031
Age (years)			0.99 (0.96–1.02)	NS

*OR* odds ratio, *CI* confidence intervals

Basal hypoNa was associated to a 2.7-fold higher probability of residual lung involvement (RALE 1) at 6 months, compared to basal normoNa. At multivariable logistic regression analysis, this association persisted even after including age, IL-6 levels and follow-up time (Table 7). Interestingly, this association was independent of serum IL-6 levels.

**Table 7:** Multivariable logistic regression analysis of factors associated with a RALE score at follow-up ≥ 1

	RALE ≥ 1 OR (95% CI)	*p*	RALE ≥ 1 OR (95% CI)	*p*
Hyponatremia	2.71 (1.29–5.66)	**0.008**	2.66 (1.12–6.31)	0.026
Follow-up (months)			1.04 (0.83–1.31)	NS
Age (years)			1.01 (1.01–1.08)	0.008
IL-6 levels (pg/mL)			1.005 (0.99–1.015)	NS

*OR* odds ratio, *CI* confidence intervals

## Discussion

We have previously shown that in patients with COVID-19 infection, hyponatremia at hospital admission (basal hypoNa) is associated with a more unfavorable outcome compared to normonatremic ones [14, 16]. In the present study, we initially considered the same cohort of patients, in order to determine whether basal hypoNa might have a clinical impact at follow-up. Among the 361 patients included in our previous study [16], a total of 172 patients were excluded for different reasons (as detailed in “Materials and methods”). Therefore, the clinical charts of 189 patients were examined.

In this subcohort of patients, the main differences between normonatremic and hyponatremic patients, which had been reported in our previous study [16], were confirmed. Specifically, patients with basal hypoNa had a lower PaO_2_/FiO_2_ ratio and higher IL-6 levels at admission than patients with normoNa. HypoNa was also associated with a higher prevalence of NIV and transfer to ICU.

In this study, we added an analytical evaluation of the chest radiographic findings of SARS-CoV-2 patients at the time of hospitalization. The RALE score has been proposed a few years ago as a numeric scoring system aiming to analyze the extent and density of alveolar opacities on chest radiographs [28]. We found that the RALE score in hospitalized patients with basal hypoNa was significantly higher than in those with normoNa and was inversely correlated with the PaO_2_/FiO_2_ ratio. Furthermore, after adjustment for age, both the presence of hypoNa and serum IL-6 levels were associated with a worse lung involvement at a multivariate logistic regression analysis. These findings are in keeping with our previous observation about the presence of a direct correlation between serum [Na^+^] and the PaO_2_/FiO_2_ ratio and of an inverse correlation between serum IL-6 and the PaO_2_/FiO_2_ ratio in COVID-19 patients [16]. The so-called cytokine storm proved to have an important role in determining an hyperinflammation syndrome in COVID-19 infection and it may contribute to a fatal outcome. IL-6 is one of the main cytokines involved in this pathology [21]. The RALE assessment has been found to be associated with a worse outcome in different conditions, such as ARDS [28, 31], refractory cardiogenic shock and refractory cardiac arrest [32]. Interestingly, in recent studies the RALE score has been indicated among the variables associated with a higher risk of death in COVID-19 patients [29, 30, 33, 34].

With regard to follow-up data, when we considered the 189 patients as a whole, we found that serum [Na^+^] and the PaO_2_/FiO_2_ ratio significantly increased at the 6-month evaluation, compared to the respective values at hospital admission. Conversely, IL-6 levels and RALE score decreased. These findings were expected in patients who survived the acute infection. Yet, it is interesting to note that serum [Na^+^] increase over time was paralleled by the resolution of the disease and the improvement of other indicators of severity.

Noteworthy, the main finding of this study is represented by the observation that some laboratory data and cardiopulmonary parameters were significantly different in COVID-19 patients with basal hypoNa at follow-up. Among lab parameters, serum levels of LDH, a marker of cell damage, and of fibrinogen, a marker of inflammation, were significantly higher at the last observation in patients with basal hypoNa, although within the normal range. Similarly, in this group of patients, the levels of serum troponin T and NT-ProBNP, which are indicators of cardiac damage, were higher. All these laboratory parameters have been associated with a worse outcome in COVID-19 infection in meta-analyses studies [35, 36].

With regard to echocardiographic parameters, in patients with basal hypoNa the value of TAPSE, an index of right ventricular systolic function was lower than in patients with basal normoNa. Accordingly, the pulmonary arterial systolic pressure (sPAP), the right ventricle dimension and the right ventricle/right atrium gradient (RV/RA) were higher in hyponatremic patients. These findings suggest a greater involvement of right-sided heart at follow-up in patients with basal hypoNa. Indeed, several meta-analyses have indicated that worse indexes of right ventricular function are associated with a more unfavorable outcome in COVID-19 patients [37–40].

It is worth mentioning that the echocardiographic parameters detected in hyponatremic patients are consistent with cardiac features observed in the well-recognized condition termed long COVID, which is characterized by a large spectrum of symptoms that are related to the involvement of multiple organs. Commonly reported symptoms are non-specific, i.e., fatigue (53.1%), worsened quality of life (44.1%), joint (27.3%) and chest pain (21.7%), which may determine functional dependence in routine daily activities and can induce mental health issues (anxiety, depression and post-traumatic stress disorder) [41]*.* Cardiac abnormalities commonly reported on follow-up include right ventricular systolic dysfunction, in addition to myopericarditis, and ischemia/infarction, arrhythmias, progression of coronary artery disease and aortic aneurysms, venous and arterial thromboembolic disease, sudden cardiac death [42]. A mild association between the probability of the persistence of symptoms and severity of acute illness, older age and a number of comorbidities has been identified. However, it is not completely understood who is at greater risk of developing long-COVID, so far [25]. Our data suggest that basal hypoNa may be an indicator of a different echocardiography profile at follow-up, compared to basal normoNa.

Right ventricular systolic dysfunction may originate from cardiac or pulmonary diseases or from a combination of both. Although no differences were observed in patients with basal hypoNa or normoNa with regard to pulmonary function testing, hemogasanalysis or 6 min walking test at follow-up, interestingly a significantly higher RALE score was observed in hyponatremic patients. Therefore, RALE score well discriminated patients with basal hypoNa or normoNa both at admission as well as at follow-up, thus indicating the persistence of a more severe pulmonary involvement in the first group. Admittedly, it is reasonable to hypothesize that the right-sided heart involvement in these patients is secondary to a more severe pulmonary damage. Persistent pulmonary alterations are also among the features observed in long COVID. Follow-up studies of COVID-19 survivors showed pulmonary radiological abnormalities and functional impairments at 3 [43, 44] and 6 months post-hospital discharge [45].

In our study, we also determined by a multivariate regression logistic analysis that basal hypoNa is associated with a 2.71 greater risk of an abnormal RALE score at follow-up. This finding was independent of serum IL-6 levels. It is on particular interest the absence of interference of IL-6 at follow-up, considering its influence on the RALE score at hospital admission, as we have shown here, the aforementioned role of this cytokine on the inflammatory storm in COVID-19 infection [21] and its short-term unfavorable prognostic role [14, 16, 21]. Therefore, we can conclude that basal hypoNa, yet not higher IL-6 levels, maintains its role in COVID-19 patients as an indicator of a more severe disease, with altered cardiologic and pulmonary profiles at follow-up.

In view of these findings, we thus suggest to consider basal serum [Na^+^] as a parameter of clinical importance both in the short term and at follow-up in COVID-19 patients. Admittedly, a serum [Na^+^] determination can be readily available in hospitalized patients. We have to say that the association between basal hypoNa and a pathologic RALE score at follow-up was not independent of age. Nevertheless, this finding does not alter the role of hypoNa in COVID-19 disease, if we consider that the incidence of this electrolyte alteration is known to increase with age [46].

In summary, to our knowledge, in this study, we have demonstrated for the first time that a low serum [Na^+^] at admission in patients with COVID-19 infection is associated with the presence of laboratory and imaging parameters indicating a greater pulmonary and right-sided heart involvement at follow-up. Admittedly, these findings reinforce the recommendation that hyponatremia is promptly recognized and appropriately corrected also in these patients that so heavily challenged health systems worldwide in the last couple of years.

## Supplementary Information

Below is the link to the electronic supplementary material.

Supplementary file1 (DOCX 13 KB)
