# Supplementary material for: Relationship between hyponatremia at hospital admission and cardiopulmonary profile at follow-up in patients with SARS-CoV-2 (COVID-19) infection
Source: J Endocrinol Invest. 2022 Oct 25;46(3):577–86. doi: 10.1007/s40618-022-01938-9 (PMC9595583; doi:10.1007/s40618-022-01938-9)
Supplement: Supplementary file 1 — Supplementary file1 (DOCX 13 KB) [file 40618_2022_1938_MOESM1_ESM.docx]

| **Supplementary Table 1** | |
| --- | --- |
| Complete Blood Count | NT-Pro-BNP |
| Serum Creatinine | D-Dimer |
| Sodium | Antithrombin |
| Potassium | Protein C And Protein S |
| Magnesium | Interleukin (IL)-6 |
| Alanine Aminotransferase | IL-8 |
| Aspartate Aminotransferase | Il-1beta |
| Creatine Phosphokinase | Il-10 |
| Lactate Dehydrogenase | Tumor Necrosis Factor Alpha |
| High-Sensitivity C Reactive Protein | Ferritin |
| High Sensitivity Troponin T | Fibrinogen |

**Supplementary Table 1. Peripheral Blood Sample items.**
